# Artery-to-Fistula Diameter Ratio as a Predictor of Early Re-Occlusion of Immature Right Radio-Cephalic Arteriovenous Fistula after Primary PTA

**DOI:** 10.3390/diagnostics12092059

**Published:** 2022-08-25

**Authors:** Moo-Jun Kim, Hojoon Ko, Suyeon Han, Eu-Jin Lee, Young-Rok Ham, Kang-Wook Lee, Dae-Eun Choi, Jin-Ah Shin, Ki-Ryang Na

**Affiliations:** 1 Nephrology, Chungnam National University Hospital, Daejeon 35015, Korea; 2 Nephrology, School of Medicine, Chungnam National University, Daejeon 35015, Korea; 3 Departement of Medical Science, Chungnam National University, Daejeon 35015, Korea

**Keywords:** arteriovenous fistulas, hemodialysis, immature, intervention, percutaneous transluminal angioplasty

## Abstract

Percutaneous transluminal angioplasty (PTA) is widely performed for arteriovenous fistula (AVF) that fails to mature after initial formation. We observed that some immature AVFs re-occlude earlier than others. We sought to investigate the predictors for early post-intervention failure of immature fistulas after primary PTA. We retrospectively reviewed the records and angiographic images of patients who had immature fistulas and thereby received PTA between 2013 and 2019 at our center. We investigated the short-term post-intervention outcomes of the patients within 90 days post-PTA. Patients who had re-occlusion within the period were defined as the early failure group and the rest as the patent group. We investigated factors associated with early failure. There were 80 eligible patients with 22 brachio-cephalic (BC) and 58 radio-cephalic (RC) AVFs. The median age of the patients was 64 years [range, 38–87]. There were 51 (63%) males and 29 (36%) females. Among the 58 RC AVFs, 10 (17%) patients had early failure. Logistic regression analysis showed that a larger artery to fistula (A/F) diameter ratio was the sole independent predictor of early failure after primary PTA (odd ratio 2.29 [1.023–5.147], *p* value = 0.044). Although further studies on a larger scale are required to confirm the clinical significance, a larger A/F diameter ratio was a potential predictor of early re-occlusion in immature fistulas after primary PTA.

## 1. Introduction

Hemodialysis is a key treatment for renal failure that uses a machine to send the patient’s blood through a filter, called a dialyzer, outside the body. Vascular access to hemo-dialyzer is a hemodialysis patient’s lifeline because it makes life-saving hemodialysis treatments possible. Successful maintenance of an arteriovenous fistula (AVF) is one of the key determinants of quality of life in patients with end-stage renal disease (ESRD) receiving hemodialysis (HD). Native AVFs are more advantageous than prosthetic arteriovenous grafts (AVGs) because they possess lower infection rates and better patency [1]. The National Kidney Foundation Kidney Disease Outcome Initiative (NKF K/DOQI) recommends AVF as the vascular access of choice [2]. AVFs, however, have a risk of non-maturation resulting from poor inflow of feeding artery, thrombosis or stenosis [3]. The maturation of an AVF is defined as a status that can be cannulated for HD [4]. AVFs with a draining vein measuring at least 4–6 mm in diameter with a depth no greater than 5–6 mm and blood flow of 500–600 mL/minute are likely to be mature and adequate for dialysis [5]. Hand exercise is performed to mature immature AVF, but in some cases, it still does not mature effectively. Indeed, Fontsere et al. reported that non-significant differences in clinical or ultrasonographic maturation were seen between exercise and control groups [6].

For those AVFs that fail to mature or occluded due to stenosis or thrombosis, a salvage percutaneous transluminal angioplasty (PTA) is a procedure that can open up a blocked blood vessel using a small, flexible plastic tube or catheter with a “balloon” at the end and therefore an effective modality. For the outcomes of immature fistulas after salvage angioplastic intervention, the location and size of the stenosis at the initial PTA [7] have been reported as associated factors for post-intervention patency. 

In this study, we evaluated the first 90-day post-intervention outcomes of patients with immature fistulas who had their first PTAs. Some had shorter post-intervention patency than others. We sought to investigate the predictors of early post-intervention failure of immature fistulas after PTA to contribute to the further management and prevention of AVF occlusion. 

## 2. Materials and Methods

### 2.1. Patients

**Figure 1:**
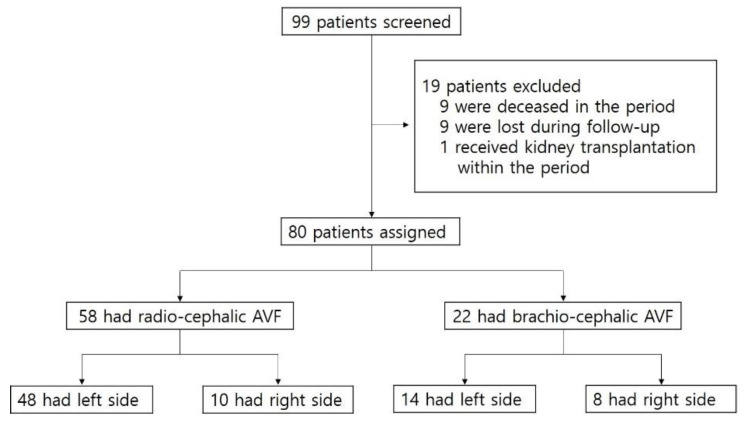
Patient assessment flow chart. AVF, arteriovenous fistula.

We retrospectively reviewed the records and angiographic images of 99 patients who had immature fistulas and thereby received PTA in the period between February 2013 and March 2019 at Chungnam National University Hospital. Among the 99 patients, 19 were excluded for the following reasons: 9 deceased in the period, 9 were lost during follow-up, and 1 received kidney transplantation within the period. Of the 80 eligible patients, 22 had brachio-cephalic (BC) AVF and 58 had radio-cephalic (RC) AVFs (Figure 1).

Information collected from the medical records review included age, race, gender and the presence of various comorbid conditions, such as hypertension, diabetes, coronary artery obstructive disease (CAOD), and peripheral artery obstructive disease (PAOD). Data regarding technical aspects of the primary PTA were obtained using data abstraction forms, which were completed by the operators immediately after each procedure. In addition, by reviewing the radiographic images recorded during the PTA, we obtained data including the date of arteriovenous fistula operation, the date of intervention, the site of the stenotic lesion, the angle of anastomosis, type and size of vessels used, degree of stenosis, and the diameters of the vessels (i.e., artery, vein and the primary AVF). 

This study was conducted according to the ethical standards laid down in the 1964 Declaration of Helsinki and its later amendments. This study was approved by the international review board of Chungnam National University Hospital (IRB No. 2015-12-025). 

### 2.2. Variables and Definitions

An immature fistula was defined as failure of HD initiation within 12 weeks post-formation (AVF formation) or re-occlusion within 4 weeks after HD initiation. We investigated 90-day outcomes following the PTA. Those who had recurrent occlusion at the same site of the PTA within 90 days of the intervention were defined as having early failure. The rest were defined as the patent group. The age of the fistula at intervention was defined as the time period from the fistula initiation and the primary PTA. 

**Figure 2:**
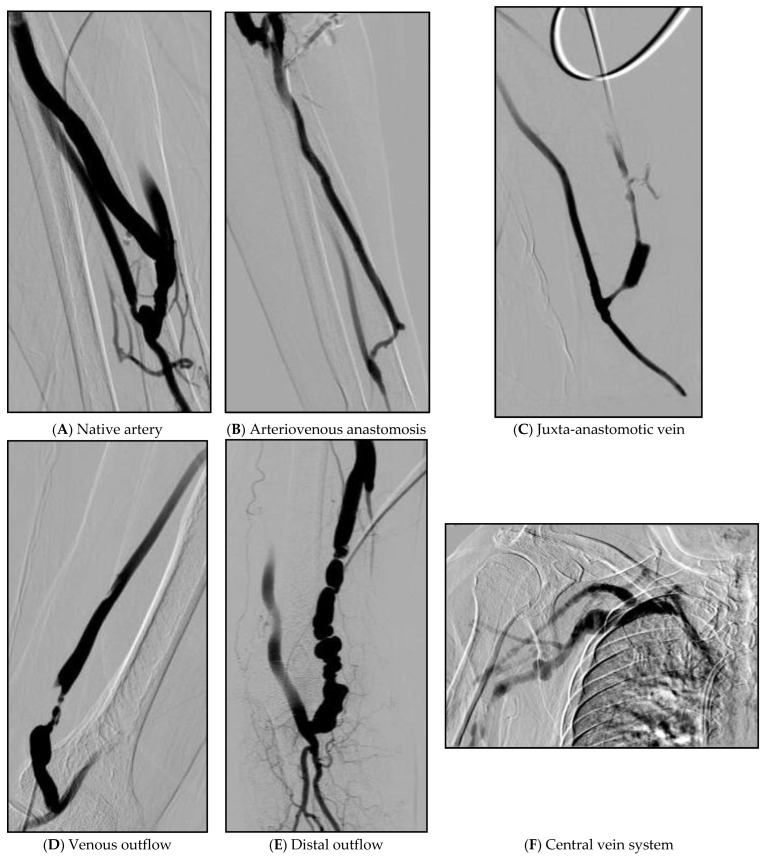
Example figures of the location of lesions. (**A**) About 70% of partial stenosis in the area above the anastomosis site of the radial artery. (**B**) Vein is narrowed by 80% from the upper part of the anastomosis site to the portion of the cephalic vein that curves almost at right angles. (**C**) About 90% of stenosis at the juxta-anastomosis site. (**D**) A segment with a length of 3 cm from 4 cm above the right brachio-cephalic AVF anastomosis site shows about 98% string-like stenosis. (**E**) Multiple web-like stenosis was observed in a beaded pattern as a 6 cm long segment from the right superior to the anastomosis of the brachio-cephalic AVF. (**F**) 70% stenosis with collateral to the right subclavian vein.

As variables, we included the reported predictors of AVF maturation, such as vein diameter [8], arterial diameter [9], and anticoagulant doses [10]. The artery to fistula diameter ratio (A/F ratio) was calculated as the diameter ratio of the native artery to that of the immature fistula. Stenotic lesions were grouped according to the stenotic sites as follows: native artery, anastomosis site, juxta-anastomotic vein (less than 2 cm from anastomosis site), venous outflow (more than 2 cm from anastomosis site), distal outflow (define as above elbow joint for radio-cephalic fistulae, above mid humerus for brachial fistulae), and central veins. Cases with more than 1 stenosis were defined as multiple stenotic. Venous stenosis was defined as venous luminal occlusion of more than 50% and grouped as follows = 50–74%, 75–89% and 90–100% (Table 1). Example figures of the location of the lesions are attached below (Figure 2).

### 2.3. Measurements

**Figure 3:**
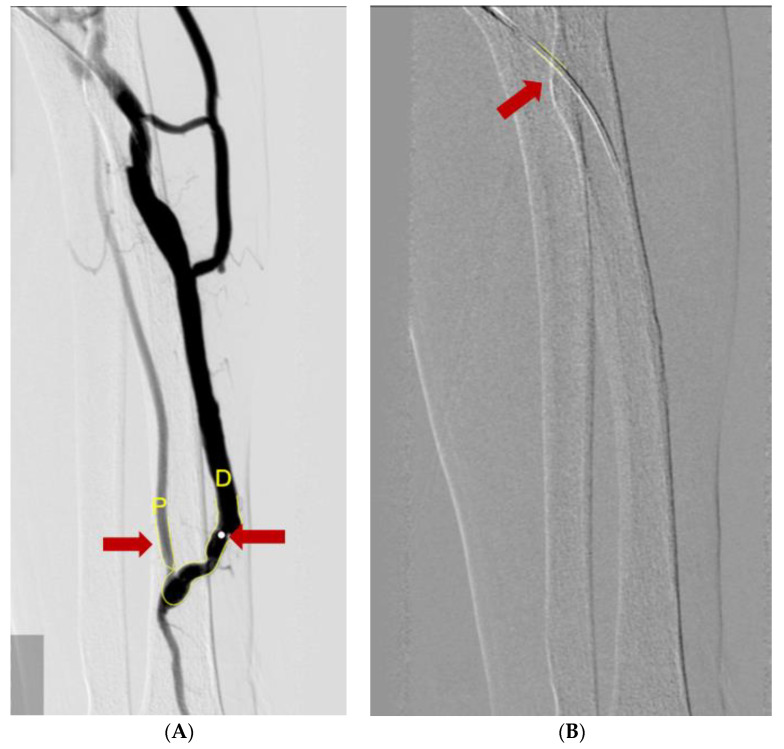
Measuring diameters of vessels using the Cardiovascular Angiography Analysis System. (**A**) The diameter of the catheter (red arrow) was used for the calibration reference. (**B**) A/F ratio was measured by ratio of diameter of radio-cephalic artery (red arrow and yellow letter P) and AVF vessel (red arrow and yellow letter D).

To measure the vessel diameters, we used a computer-assisted automatic contour detection technique (CAAS 5.9; Pie Medical Imaging, Maastricht, The Netherlands). The outer diameter of the PTA catheter filled with the contrast was considered the calibration standard (5Fr or 6Fr). We measure the diameter of the vein and artery after the stenotic site. To compensate for the calibration errors, we used the A/F ratio coefficient in the statistical analysis rather than the diameter itself (Figure 3).

### 2.4. Statistical Analysis

PASW Statistics ver. 22.0 software (IBM Co., Armonk, NY, USA) was used, and *p*-values < 0.05 were considered significant. Continuous data were analyzed as medians with ranges and categorical data as proportions and percentages. An unpaired *t* test was used for continuous variables, and chi square was used for categorical variables to compare the RC and BC AVF groups. ROC-AUC curve analysis was used to obtain the best cut-off values of A/F ratio in early failure versus patent groups with immature AVF of RC type. We used logistic regression analysis to investigate the risk factors associated with the early failure group. The RC-AVF population could be stratified on the basis of a threshold value of the artery-to-fistula diameter ratio in a categorical binary variable. Then, we compared the early failure rate with the artery-to-fistula diameter ratio either below group or over the threshold value group. 

## 3. Results

There were 80 eligible patients who had immature fistulas and were followed up for the study period. The number of lesions of stenosis was most common at 1 site (52.5%). (Table 1) As for location of lesions, the juxta-anastomotic vein was the most common with 55 (66.3%), followed by venous outflow with 32 (40.0%) (Table 1). There were 22 BC and 58 RC AVFs (Table 2). The median age of all eligible patients was 64 years [range, 38–87]. The median age of RC AVF patients was 61 years [range, 38–85] and the median age of BC AVF patients was 68 [range, 48–87], respectively. Among the comorbid conditions, hypertension was the most common in 60 (75%) patients, followed by 56 (70%) diabetes mellitus, 24 (30%) CAODs, and 4 (5%) PAODs. We investigated whether the target patients were taking aspirin, clopidogrel, warfarin or cilostazol, and no significant *p*-value was derived. (Table 2). Most patients had left-sided AVFs, 62 (77.5%) and some had right-sided AVFs, 18 (22.5%). The median age of fistula at primary PTA was 78 months [30–1404], and the median A/F ratio was 0.69 [0.2–1.34]. Most patients had 80% [50–100] occlusion, and 38 patients had multiple stenosis lesion (38/80, 47.5%). Comparing the data of RC (n = 58) and BC AVF (n = 22), RC group had older age of fistula (86 months [30–1404] than BC group (63 months [33–295]) at the primary PTA, fewer events of CAODs (22.4% in RC group than 50% in BC group) and smaller A/F ratio (0.62 [0.20–1.22] in RC group than 0.91 [0.70–1.34] in BC group). 

For the comparison analysis between the early failure group and patent group, BC AVF were not included due to the scantity which did not permit statistical analysis. Among the 58 RC AVFs, 10 (17%) patients were grouped as ‘early failure’ and the remaining 48 (83%) were considered as ‘patent’. Clinical characteristics and data regarding the PTA of early failure versus patent groups with immature AVF of the RC type are summarized in Table 2. 

Between the total group and RC group, the age was similar at 64 and 61, respectively and the comorbidity ratio was also similar without any significant difference. In the early group and the patent group medications, the occurrence of early failure was significant when cilostazol was taken (*p* value = 0.027, Table 3). The C-reactive protein (CRP) level was also not significantly different between the early failure group (0.30 [0.10–0.70] and patent group (0.41 [0.10–10.00] at the time of the first intervention.

**Figure 4:**
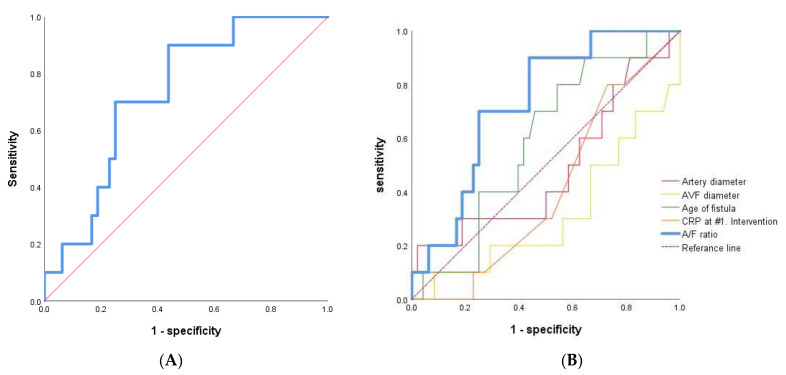
ROC-AUC curve analysis. (**A**) A/F ratio as the sole independent predictor of early AVF failure after primary PTA in early failure versus patent groups with immature AVF of RC type (n = 58). (**B**) The traditional factors, including age of fistular, artery diameter, AVF diameter, CRP and A/F ratio were analyzed.

ROC-AUC curve analysis was done, generating cut-off values for A/F ratio of 0.690 in early failure versus patent groups with immature AVF of RC type (AUC = 0.731, *p* value = 0.022, 95% CI = 0.585–0.878, Figure 4A). The ROC-AUC of traditional factors, including age of fistular, artery diameter, AVF diameter, and CRP at 1st intervention, had low AUC and statistically insignificant values compared to A/F ratio (Figure 4B).

On multivariate logistic regression analysis, a larger A/F ratio was the sole independent predictor of early AVF failure after primary PTA (0.71, early failure group vs. 0.60, patent group; odds ratio 0.167 [0.030–0.939], *p* value = 0.042) (Table 4). 

**Figure 5:**
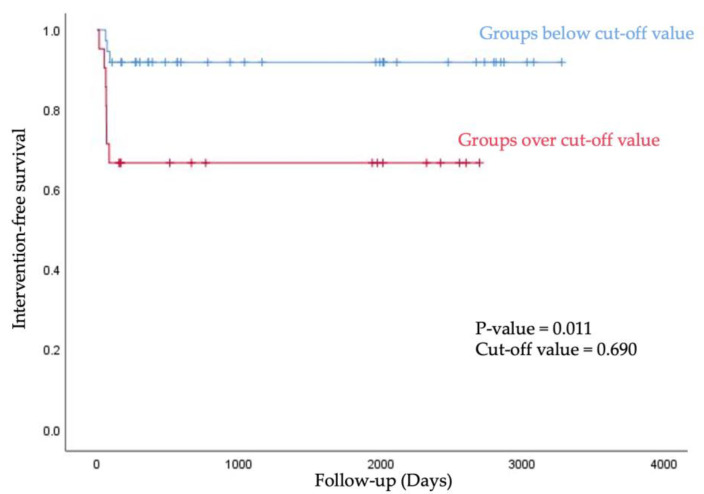
For intervention-free survival, the Kaplan–Meier curve was obtained after stratification into below group and over group based on the cut-off value of A/F ratio.

We stratified into below group and over group based on the cut-off value of A/F ratio, and then analyzed the intervention-free survival rate using the Kaplan–Meier curve. Groups below the 0.690 value of A/F ratio showed longer intervention-free survival within 90 days after first AVF use (*p* value = 0.011, Figure 5).

## 4. Discussion

ESRD patients in need of HD have only a limited number of available vessels for the creation of AVF [11]. Prediction of the outcomes of AVFs post-PTA, therefore, is worth investigation. In this study, we were able to identify A/F ratio as a possible predictor of early failure in immature fistulas after primary intervention.

Currently, there is no consensus on fistula maturation [8]. Dember and colleagues identified functional maturation of AVF as fistula suitability with two needles to maintain an optimal dialysis flow rate of ≥300 mL/min during eight of 12 dialysis sessions [12]. The NKF K/DOQI glossary defines fistula maturation as “the process by which a fistula becomes suitable for cannulation” [8,13,14]. Lauvao et al. defined fistula maturation as the time from access creation to a time an assessment by a vascular surgeon and nephrologist determines a fistula can be cannulated [8]. Fila et al. defined immature fistula as an AVF that thrombosed before the first cannulation or did not reach the functional status, patent or not [11]. Here, we defined fistula maturation as the failure of HD initiation within 12 weeks post-operation or an AVF requiring primary PTA within 4 weeks after HD initiation.

The importance of vessel characteristics in the successful prediction of AVFs has been recognized by many authors [4,8,15,16,17,18,19,20]. Wong et al. [17] reported that any AVFs with a vein with a diameter of 1.6mm or less would fail. Moreover, Ferring et al. reported a greater rate of maturation success (76% vs. 16%) in AVFs made with veins of diameter larger than 2 mm compared to those with 2 mm or less [18]. Furthermore, the KDOQI recommended that AVF should be created when the luminal diameter of the artery is at least 2 mm and of the vein at least 2.5 mm [2]. Despite a body of literature on the predictors of primary AVF failure, sparse studies have reported on the predictive vascular factors associated with re-stenosis of immature fistulae after PTA.

In this study, we sought to investigate factors that may affect early post-PTA stenosis of immature fistulae. Higher A/F ratio was the sole independent predictor of early AVF failure after primary PTA. In other words, as the size difference between the diameter of the artery and the immature AVF became smaller (0.71, early failure group vs. 0.60, patent group), the likelihood of early re-occlusion increased. This finding may be contrary to the previous study by Rezapour and colleagues [4], which reported that maturation time is shorter when the diameters of the vein and artery are close to one another. To this end, we contemplate that the vein of the immature fistula may have had less plasticity to thicken and/or dilate than its native state. Therefore, greater shear stress and increased transmural pressure due to decreased flexibility of the immature fistulae may have contributed to neointimal hyperplasia and adverse vascular remodeling. Several studies have already shown that high shear stress at the stenosis can activate platelets and von Willebrand factor, and the shear micro-gradient across the stenosis promotes platelet aggregation [21,22], and a higher A/F ratio may be highly likely to be related. Kim et al. reported better efficacy of balloon-assisted maturation in immature autogenous radio-cephalic AVFs for hemodialysis [23]. It is expected that if patients with a high A/F ratio in immature AVFs are selected and when PTA is performed in advance for maturation, early re-occlusion can be prevented.

We must acknowledge that our study has several limitations. This is a single-centered and retrospective review that relies on available patient information, and it is vulnerable to availability. However, since the results such as A/F ratio are objective values extracted through angiography and we used the A/F ratio coefficient in the statistical analysis to compensate for the calibration errors, the difference according to the center is thought to be insignificant. Our group size is limited and small; therefore, only patients with radio-cephalic fistulas could be included in the analysis. Furthermore, our definition of fistula maturation is limited to our clinical assessment after fistula placement. We may also expect better investigation if we measured the diameters of the native veins prior to the PTA.

## 5. Conclusions

In this study, a larger A/F ratio was a possible predictor of early re-stenosis of immature fistulas after primary PTA. Based on the results of this study, it may be possible to reduce AVF stenosis in the future if arteries and veins that can reduce A/F ratio are selected during arteriovenous fistula surgery.

Although further studies are warranted to verify its clinical significance, it is tempting to suggest that patients with a larger A/F ratio should be more alertly followed up for early re-occlusion following PTA. It is hoped that this study will establish a diagnostic tool that can detect and treat arteriovenous fistula malfunctions in patients undergoing hemodialysis at an earlier stage.

## Figures and Tables

**Table 1:** Angiographic findings of immature fistula at primary PTA.

	Category	N (%)
Degree of stenosis		
Stenosis	90–100%	35 (43.7)
	75–89%	19 (23.8)
	50–74%	26 (32.5)
Number of lesions	1 site	42 (52.5)
	2 sites	28 (35.0)
	≥3 sites	10 (12.5)
Location of lesions	Native artery	11 (13.8)
	Arteriovenous anastomosis	15 (18.8)
	Juxta-anastomotic vein	53 (66.3)
	Venous outflow	32 (40.0)
	Distal outflow	17 (21.3)
	Central venous system	2 (2.5)

**Table 2:** Patient characteristics and data regarding immature fistulas.

Characteristics	Total (N = 80)	RC AVF (N = 58)	BC AVF (N = 22)	*p* Value
Age (years)	64 [38–87]	61 [38–85]	68 [48–87]	0.100
Gender, n (%)				
Female	29 (36.3)	19 (32.8)	10 (46)	0.213
Comorbidity, n (%)				
Diabetes (%)	56 (70.0)	42 (72.4)	14 (63.6)	0.307
Hypertension (%)	60 (75.0)	43 (74.1)	17 (77.2)	0.509
CAOD (%)	24 (30.0)	13 (22.4)	11 (50.0)	0.018
PAOD (%)	4 (5.0)	2 (3.4)	2 (10.0)	0.303
Medication (%)				
Aspirin (%)	30 (37.5)	21 (36.2)	9 (40.9)	0.797
Clopidogrel (%)	11 (13.8)	8 (13.8)	3 (13.6)	0.999
Warfarin (%)	1 (1.3)	1 (1.7)	0 (0)	0.999
Cilostazol (%)	4 (5.0)	3 (5.2)	1 (4.5)	0.999
Fistula characteristics				
Right side, n (%)	18 (22.5)	10 (17.2)	8 (36.3)	0.066
Age of fistula	78 [30–1404]	86 [30–1404]	63 [33–295]	0.018
A/F ratio	0.69 [0.20–1.34]	0.62 [0.20–1.22]	0.91 [0.70–1.34]	0.000
Degree of stenosis %	80 [50–100]	80 [50–100]	80 [50–100]	0.544
Multiple stenosis lesion, n (%)	38 (47.5)	27 (46.6)	11 (50.0)	0.566
Early failure	14 (17.5)	10 (17.2)	4 (18.1)	0.606

**Table 3:** Characteristics of early failure versus patent groups with immature AVF of the RC type.

Variable	Total	Group		
		Early (<90 Days)	Patent	*p* Value
Number of patients	58	10 (17)	48 (82)	
Age (years)	61.0 [38.0–85.0]	64.5 [55.0–81.0]	60.5 [38.0–85.0]	0.380
Gender, n (%)				
Female	19 (32)	2 (20)	17 (35)	0.472
Comorbidities, n (%)				
Diabetes (%)	42 (72)	5 (50)	37 (77)	0.119
Hypertension (%)	43 (74)	7 (70)	36 (75)	0.708
CAOD (%)	13 (22)	2 (20)	11 (22)	0.999
PAOD (%)	2 (3)	0 (0)	2 (4)	0.999
Medication (%)				
Aspirin (%)	6 (10.3)	3 (30.0)	3 (6.3)	0.057
Clopidogrel (%)	3 (5.2)	1 (10.0)	2 (4.2)	0.439
Cilostazol (%)	2 (3.4)	2 (20.0)	0 (0)	0.027
Warfarin (%)	0 (0)	0 (0)	0 (0)	-
Fistula characteristics				
Right side fistula, n (%)	10 (17)	2 (20)	8 (16)	0.999
Age of fistula	88.5 [32.0–1404.0]	96.0 [43.0–729.0]	83.5 [32.0–1404.0]	0.965
A/F Ratio	0.62 [0.20–1.22]	0.71 [0.54–1.22]	0.60 [0.20–1.00]	0.027
Artery diameter	3.33 [1.00–5.75]	3.12 [1.5–5.75]	3.4 [1.0–5.3]	0.809
AVF diameter	5.21 [2.10–9.00]	4.66 [2.10–7.27]	5.38 [3.07–9.00]	0.057
Degree of stenosis %	80.0 [50.0–100.0]	80.0 [50.0–100.0]	80.0 [50.0–100.0]	0.321
Multiple stenosis lesion, n (%)	27 (46)	5 (50)	22 (45)	0.502
CRP at #1. Intervention	0.30 [0.10–10.00]	0.30 [0.10–0.70]	0.41 [0.10–10.00]	0.331

**Table 4:** Results of multiple logistic regression in early failure versus patent groups with immature AVF of the RC type.

Variable	Odds Ratio	95% CI	*p* Value
Gender	0.403	0.059–2.767	0.355
Age (years)	1.042	0.965–1.125	0.355
Diabetes	0.508	0.084–3.055	0.298
Hypertension	1.055	0.172–6.467	0.459
CAOD	0.768	0.073–8.017	0.954
PAOD	-	-	-
Aspirin	0.786	0.122–5.058	0.786
Clopidogrel	1.165	0.077–17.634	1.165
Cilostazol	0.310	0.025–3.840	0.310
Warfarin	-	-	-
A/F ratio	0.167	0.030–0.939	0.042
